# Worldwide Age at Onset of Female Breast Cancer: A 25-Year Population-Based Cancer Registry Study

**DOI:** 10.1038/s41598-019-50680-5

**Published:** 2019-10-01

**Authors:** Ettore Bidoli, Saverio Virdone, Mokhtar Hamdi-Cherif, Federica Toffolutti, Martina Taborelli, Chiara Panato, Diego Serraino

**Affiliations:** 1 Unit of Cancer Epidemiology, Centro di Riferimento Oncologico di Aviano, IRCCS, via Gallini 2, 33081 Aviano, PN Italy; 2 Faculty of Medicine and Cancer Registry, University of Setif, Setif, Algeria; 3 Friuli Venezia Giulia Cancer Registry, IRCCS Centro di Riferimento Oncologico, Aviano, Italy

**Keywords:** Health care, Epidemiology

## Abstract

A higher frequency of early onset female breast cancers (BC) has been observed in low/middle income countries than in high income countries. We quantified the role of population ageing to this pattern using data from all population-based cancer registries (CRs) worldwide. Patients’ median age at BC onset and that of the general population were extracted for CRs listed in volumes VI (1983–1987 years) through XI (2008–2012 years) of Cancer Incidence in Five Continents. Their association was assessed at cross-sectional level by linear regression model and longitudinally considering 25-year ageing of the population in long-standing CRs listed at the beginning and at the end of the study. During 2008–2012, each one-year increase of population ageing was associated with a nearly ½ year increase of age at BC diagnosis. Population demographics explained forty-two percent of the age variance for BC. In 1983–1987, long-standing CRs with a median age at BC below age 61.8 years showed an increase of age at BC after 25-years. Worldwide, age at BC diagnosis essentially reflected the median age of the population. Changes in BC detection methodology likely lessened this association. Nevertheless, the elevated absolute number of BCs in young populations deserves strategies of BC prevention.

## Introduction

Breast cancer (BC) in women is the most frequent cancer diagnosed worldwide, in any of the five continents, with an estimated 2.1-million new cases during 2018^1^. A higher frequency of early onset female BC has been observed in low/middle income countries (LMIC) than in high income countries (HIC), with an observed median age for female BC diagnoses in HIC about a decade higher than in LMIC, such as some Arab or Asian countries^2–16^. This observation has indicated the need to lower the age of entry into mass BC screening programs in LMIC^8^. Differing median ages at BC onset among countries have been mainly attributed to the different shapes of the underlying population pyramids^6,13,17–19^, and/or to low BC incidence rates in older women in LMIC^20^. In fact, in LMIC, the population pyramids are skewed toward younger age groups, showing a cone-shaped pattern whereas HIC show an urn pattern^21^. The number of incident BC cases are expected to be higher where the female population is greater (i.e., young women in LMIC, and middle-aged/old women in HIC). Thus, the lower median age for BC in LMIC does not unarguably mean that younger women are more likely to have a BC.

Although younger age structure is intuitively linked to a higher frequency of young age at BC onset, the degree of this association has never been quantified^22^. First of all, to measure the extent of this association, we summarized each population pyramid by calculating the median age of the population. Then, we visualized and quantified the impact of population ageing on age at onset of BC by means of two approaches: 1) a cross-sectional analysis of the median age for BC *vs*. the median age of the corresponding population, and 2) a longitudinal analysis measuring the association between the progressive population ageing and the age for BC over a given time span.

To systematically address this issue, we took advantage of the availability of computerized BC incidence data among all cancer registries (CRs) listed worldwide by International Agency for Research on Cancer (IARC) over the 1983–2012 period to describe and quantify the association between the median age for BC cases and the median age of the underlying population at risk. For the cross-sectional approach, we examined six distinctly selected periods of registration, and for the longitudinal approach, only the long-standing CRs that had collected BC incidence over a 25-year time span, i.e. both during 1983–1987 and 2008–2012.

## Results

During the whole 2008–2012 period, our study included 2.6 million incident BC cases gathered in the considered CRs. Furthermore, in the long-standing CRs the total number of BC cases varied from 369,042 during 1983–1987 period to 672,161 during 2008–2012 period (data not shown).

**Figure 1:**
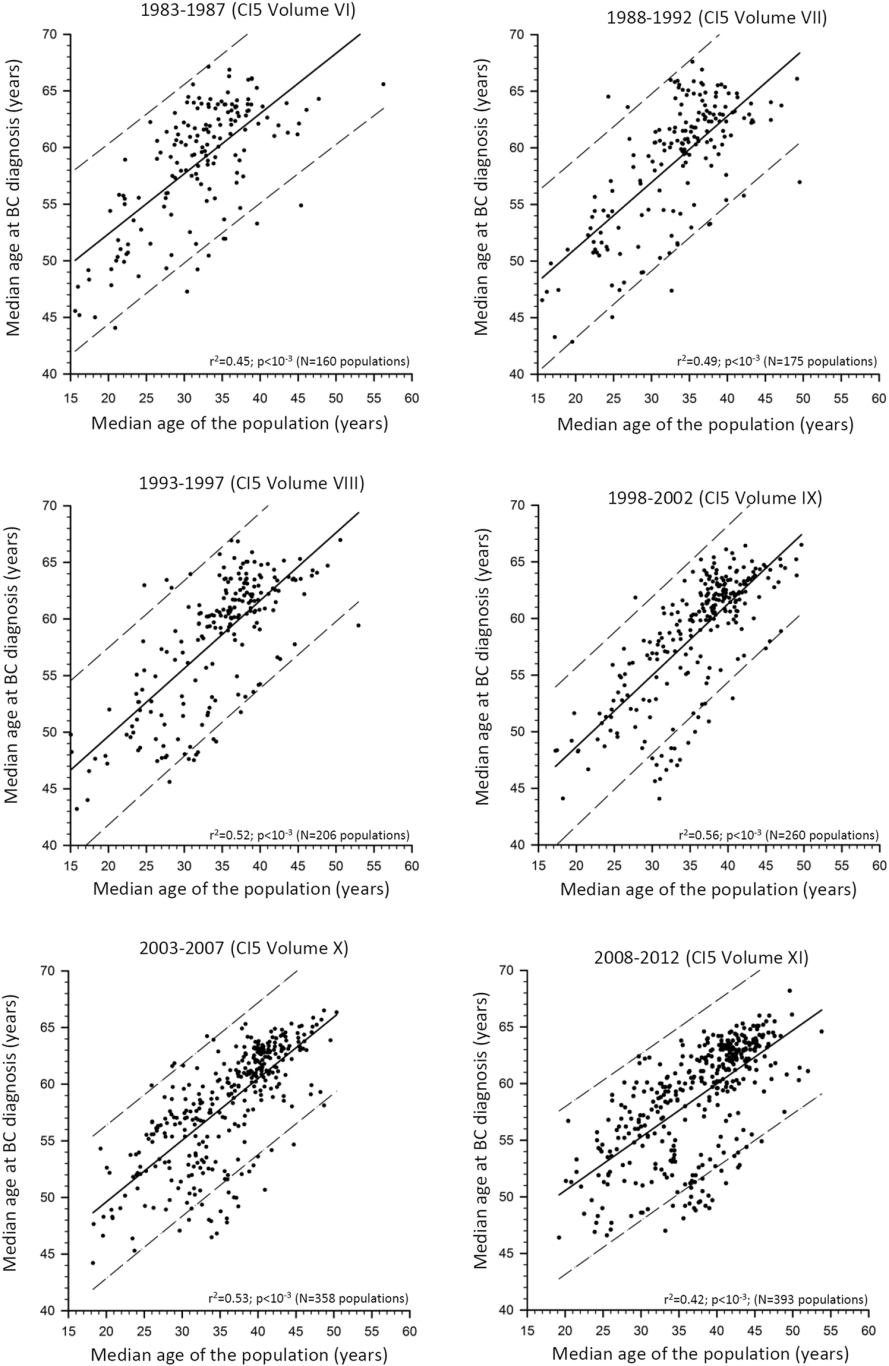
Scatter plots and linear regressions with corresponding 95% predictive intervals, of the median age of the population *vs*. the median age of breast cancer (BC) cases in the populations recorded in all cancer registries listed in the Cancer in Five Continents (CI5) volumes, according to six calendar periods.

Figure 1 shows the association between the median age for BC and the median age of the corresponding population by means of scatter plots and linear regressions, separately, for the examined six calendar periods. The intercept and the slope of the regression line, the r^2^ and the number of all listed CRs are reported in Table 1 for each calendar period. Concerning the 2003–2007 and 2008–2012 periods, data are further disentangled by the three selected broad geographical areas.

**Table 1:** Linear regression between median age of the population as a predictor of the median age at breast cancer according to six calendar periods recorded by the Cancer in Five Continents volumes. For the Volumes X and XI, cancer registries were also stratified by three main geographical areas.

Calendar period (IARC Volume)	Number of populations recorded by the Cancer Registries	Intercept	Slope of the regression line (95% Confidence Interval)	Adjusted r^2^
1983–1987 (VI)	160	41.7	0.53 (0.44–0.62)^*^	0.45^+^
1988–1992 (VII)	175	39.4	0.59 (0.50–0.67) ^*^	0.49^+^
1993–1997 (VIII)	206	37.7	0.60 (0.52–0.68) ^*^	0.52^+^
1998–2002 (IX)	260	36.1	0.63 (0.56–0.70) ^*^	0.56^+^
2003–2007 (X)	358	38.7	0.54 (0.49–0.60) ^*^	0.53^+^
*-Eastern Mediterranean and Africa*	20	*41*.*8*	*0*.*34* (*0*.*04–0*.*63*) ^*^	*0*.*18*^+^
*-Asia*	56	*43*.*3*	*0*.*26* (*0*.*15–0*.*37*) ^*^	*0*.*26*^+^
*-Americas*, *Europe*, *and Oceania*	282	*42*.*5*	*0*.*47* (*0*.*43–0*.*52*) ^*^	*0*.*62*^+^
2008–2012 (XI)	393	41.1	0.47 (0.42–0.53)^*^	0.42^+^
*-Eastern Mediterranean and Africa*	24	*44*.*7*	*0*.*26* (*0*.*03–0*.*49*)^*^	*0*.*14*^+^
*-Asia*	84	*42*.*0*	*0*.*30* (*0*.*21–0*.*39*)^*^	*0*.*33*^+^
*-Americas*, *Europe*, *and Oceania*	285	*46*.*1*	*0*.*39* (*0*.*35–0*.*42*)^*^	*0*.*63*^+^

^*^p < 0.01; ^+^probability of F-test < 0.01.

In all six calendar periods, a statistically significant direct relationship was observed between median age for BC and median age of the population. This means firstly that a younger age structure of a population is linked to a high frequency of young age at BC whereas an older age structure of a population is linked to a higher frequency of older age at BC, and secondly, that the two medians tended to follow a linear pattern in any population worldwide. In particular, in the 2008–2012 period, we observed a slope of +0.47 (95% confidence interval, CI: +0.42 to +0.53; p < 0.01 showing that each one-year increase in population ageing was associated with a nearly ½ year increase of median age at BC onset. Moreover, the r^2^ value suggested that 42% percent of the variance of the median age of BC was explained by the variance of population ageing through their linear relationship. Likewise, in the other five calendar periods the estimated slopes varied from +0.53 (95% CI: +0.44 to +0.62; p < 0.001) during 1983–1987 to +0.54 (95% CI: +0.49 to +0.60; p < 0.01) during 2003–2007, while r^2^ values ranged from 0.45 (during 1983–1987 period) to 0.53 (during 2003–2007). The association between median age of the population and median age at BC onset was further measured, separately, in the three selected geographical areas both in the 2003–2007 and 2008–2012 periods. During the 2008–2012 period the slopes were 0.26 (95% CI: +0.03 to +0.49; p < 0.01), 0.30 (95% CI: +0.21 to +0.39; p < 0.01), and 0.39 (95% CI: +0.35 to +0.42; p < 0.01) for Eastern Mediterranean and Africa, Asia, the Americas, Europe, and Oceania, respectively, while corresponding r^2^ values were 0.14, 0.33, and 0.63. Somewhat similar values emerged during the 2003–2007 period. As a sensitivity analysis, we recalculated models for the 2008–2012 period, excluding 109 cancer registries that did not fully meet all IARC quality criteria (i.e, we analyzed 284 cancer registries instead of 393). No relevant differences were noted for the slopes. Slopes were: 0.47 (95%CI: 0.42–0.53) and 0.43 (95%CI: 0.35–0.51) for the 393 CRs and the 284 CRs, respectively.

**Figure 2:**
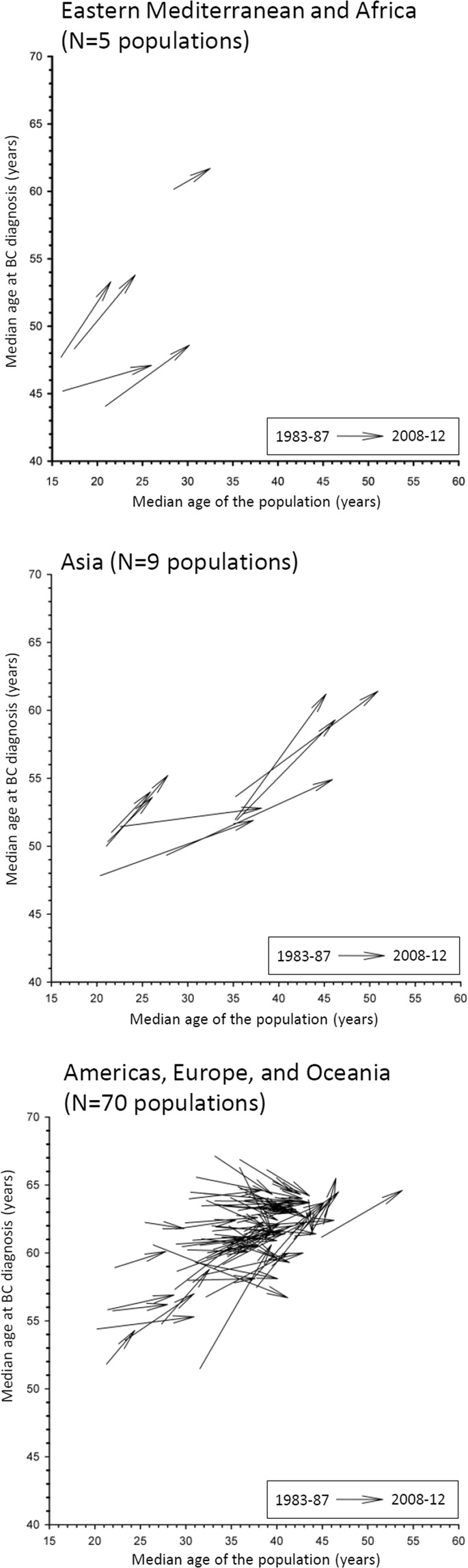
Vector plots of the median age of the population and the median age at breast cancer (BC) at the beginning (1983–87) and at the end (2008–12) of the studied period in the 84 populations of the long-standing cancer registries (CRs) listed in the Cancer in Five Continents volumes, stratified by three main geographical areas.

A 2-point line graph displayed the median age at BC according to the median age of the underlying populations of the long-standing CRs at the beginning (1983–2087) and at the end (2008–2012) of the study period, separately, in the three selected geographical areas (Fig. 2). In the long-standing CRs of Eastern Mediterranean and Africa (N = 5 populations) and Asia (N = 9 populations) all median ages for BC increased with ageing of the population during the 25-year span (median increase =+4.5 years in Eastern Mediterranean and Africa, and +5.2 years in Asia). Conversely, in the Americas, Europe, and Oceania CRs (N = 70 populations), the pattern was different during the same 25-year span. Specifically, 40% of the CRs showed a decrease in median age for BC (median of decrease = −1.1 years) and 60% showed an increase (median increase =+1.4 years) although all populations were ageing. A ROC curve analysis, including the Americas, Europe, and Oceania CRs, showed an age cut-off at BC of 61.8 years during the 1983–1987 period that predicted its decline after 25-years (i.e. in 2008–12) (not shown). The ROC curve displayed a Youden Index J of 0.61, an Area under Curve of 0.90 (95% CI:0.81–0.95), a sensitivity of 79% (95% CI: 59–92), and a specificity of 82% (95% CI:70–91). By contrast, the median age of the population during the period 1983–1987 was less predictive of the decline of age at BC after 25-years (AUC = 0.68 with a cut-off of the median age of the population equal to 35.7 years).

**Figure 3:**
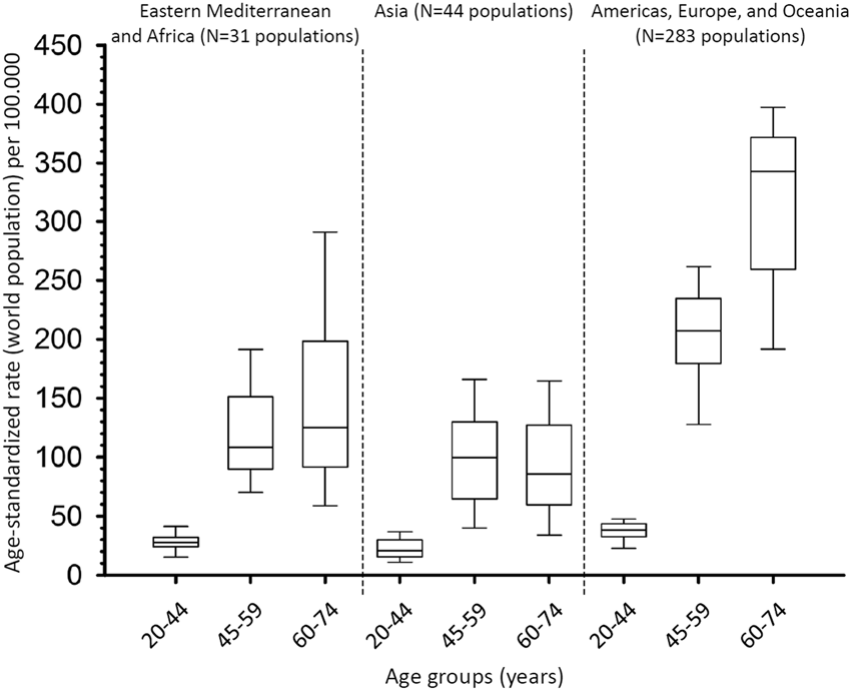
Box plot of the age-standardized incidence rates (world population) of breast cancer in the populations of all cancer registries (CRs) listed in the XI Cancer in Five Continents volume stratified by three main geographical areas and selected age groups, 2008–12.

The box-plots of the ASRs of incident BC according to the three selected geographical areas and selected age groups of the period 2008–2012 are reported in Fig. 3. The medians of the ASRs always displayed a similar pattern in CRs across age groups (i.e., the lowest rates in Asia, the intermediate rates in Eastern Mediterranean and Africa, and the highest rates in the Americas, Europe, and Oceania). As compared to the Americas, Europe, and Oceania, CRs of Eastern Mediterranean, Africa, and Asia displayed a median ASR 25–40% lower in 20–44-year age group, nearly half lower in the 45–59-year age group and around a third lower in the 60–74-year age group.

## Discussion

Our observational study, the first based on all validated worldwide CRs data gathered in 25-years, showed that median age at BC onset was proportional to the median age of the underlying population at risk. The association was independent of the geographical location of the CRs although the slopes of the linear association varied. Likewise, when examining the long-standing CRs, a median age at BC up to 61.8 years at the beginning of the study (i.e. 1983–87) tended to be associated with an increase in median age at BC onset after 25-years, whereas a median age at BC above 61.8 years was generally associated to a subsequent decrease of the age at BC onset after 25-years.

These results are broadly consistent with the findings from other epidemiological investigations conducted both in LMIC and in HIC^6,13,17–20,22^. Several plausible biological mechanisms linked to population demography, early diagnosis of BC, and to specific BC risk factors may elucidate the observed associations.

A lower median age at BC onset in some Arab and Asian countries, as compared to other countries, has been observed^3,5–9,13,15,23^, but the age distribution of the underlying populations was rarely taken into account^13,17,20^, for instance by calculating age-specific incidence rates, although ageing is linked with increasing incidence of cancers^24^. Our worldwide comparative study showed that a low median age at BC onset is a common feature of all young populations rather than a peculiarity of specific populations. The direct association between age at BC and population demographics has been previously reported in specific populations by some cross-sectional studies^17,20^, and by one longitudinal study^13^.

Moreover, in the long-standing CRs, we observed that a 25-year ageing was associated to an increase in the age at BC when this age was mostly below 61.8 years during the initial period of observation of the study, otherwise the age at BC tended to decrease. It seems likely that this pattern may be linked to the changes in the referral for early diagnosis or screening (generally recommended between 50 and 69 years of age), and to a better access to health care facilities, rather than to an abrupt change in BC risk factors during the examined 25-year span. Even if the observation of a low median age at BC alone cannot support an earlier start of mammographic screening in Arab populations^8^, the increasing ageing of these populations require the implementation of population-based BC screenings according to international recommendations^25^.

Our estimate of 42% of the variability of the age at BC onset explained by the age of the population is slightly lower than the estimate of about two thirds calculated by comparing the CRs from four Latin American countries and USA^20^. It should be noted that 63% of the variability was explained in the Americas, Oceania, and Europe while a less explained variability (14%) was observed, in our study, in the Eastern Mediterranean, Africa, and in Asia (33%). Although a different calculation methodology was used by the two studies, they supported similar conclusions. It seems likely that the unexplained variability should be ascribed to differential exposures to BC risk factors among CRs, and/or to different BC awareness and screening activities.

By examining ASRs, we observed an increasing disparity of rates with increasing age in the Eastern Mediterranean, Africa, and Asia, as compared to the Americas, Europe, and Oceania. This pattern may reflect that known BC risk factors such as parity, age at first birth, latter childbirth, breast feeding, hormonal contraceptive use, physical activity, obesity, diet, and family history of BC were unevenly distributed across age groups. Another explanation is a lack of BC incidence recording in older age groups in some CRs^20^. In any case, ageing and a trend in epidemiologic transition in young populations^21^ will increase the number of women at risk of developing BC.

Therefore, although our models tended to indicate that nearly half of BC cases were explained by population ageing, the numerical burden of young women with early BC justify public awareness campaigns towards early detection (possibly population-based), according to International directives^25^, especially in young populations regardless of income of the country of living (i.e., LMIC but also in HIC). Moreover, public awareness about BC risk factors is another key point to consider.

It should be noted that population age explained better the age variance at BC diagnosis in the Americas, Europe, and Oceania than in all other geographical areas. A possible reason for this pattern is the higher awareness raised about BC primary and secondary prevention by means of mass prevention campaigns that led to a more homogeneous behaviour concerning age at diagnosis^6^. Another possible explanation is the full epidemiologic transition raised in the Americas, Europe, and Oceania that shifted globally the burden of diseases from mainly infectious diseases to chronic non-communicable diseases^22^.

Several strengths of this investigation deserve attention. First, the present study took advantage of the worldwide cancer registration, and of long-term data gathering in specific CRs. Second, all analyzed CRs met IARC standards for validation, high-quality and comparability of the methodology of registration together with the guarantee of the complete coverage of the CRs resident population. Third, the classification of CRs by geographical area was based on previous papers and, although potentially imperfect, it improved the knowledge on the studied age patterns. Fourth, it is likely that the sample was adequately large to reduce faulty interpretation due to random variations, when analyzing CRs by main geographical areas. Moreover, the ample variability of the pyramids of age of the various CRs gave clues to the interpretation of this investigation. Fifth, the change in BC international classification of BC (IX to X revision) is unlikely to have influenced results due to the aforementioned validation of IARC.

Conversely, the study suffered from some substantial limitations, common to other observational studies. First the lack of information on BC risk factors relating to lifestyle, nutritional, reproductive habits, and medical history, and the use of screening tests limited causal inferences on unexplained variability. Further studies including the collection of individual information on screening practices are required to provide useful input to the debate on the appropriateness of lowering the age of entry into BC mass screening programs for certain populations^8^. Second, the objective of the present study was to assess the association between the median age of the population and the median age at BC onset in CRs, and we assumed a linear relationship merely by examining their scatterplot and analytically excluding the exponential model. Furthermore, all medians were extrapolated from matrices of quinquennia of age, and these estimates may be somewhat imprecise in CRs with small populations. Third, caution must be exercised in generalizing our results at country level, since, some local CRs have a small base population. The age of the population explained part of the variability of the age at BC onset, and it was observed worldwide. The remaining unexplained variability by the linear model may reflect the proportion attributable to the specific BC risk factors and to early detection that deserves careful strategies and organization in terms of prevention and early detection.

## Methods

### Data sources

An observational study was conducted using all reported incident female BC cases worldwide, occurred during the 1983–2012 period. We extracted these publicly available data from six volumes (i.e., VI-XI) of Cancer Incidence in Five Continents^26–28^. Each volume collected cancer incidence data from all population-based, country-specific, or region-specific high-quality CRs located across the world (i.e Asia, Oceania, Africa, Europe, and the Americas). Data were disentangled by age (quinquennia), sex, and populations (ethnicities) of each CR. The volumes provided data for each CR typically for a quinquennia (volume VI:1983–1987, VII:1988–1992, VIII:1993–1997, IX:1998–2002, X:2003–2007, and XI:2008–12). Not all CRs were able to provide data for the whole period covered by each volume but a minimum three-year consecutive series was the inclusion requirement^26^. To avoid data duplicates, or the use of estimates of cancer incidence, we excluded the CRs derived by summing data from multiple populations, multiple CRs, or obtained from regional cancer estimations. The number of these unique populations increased from 160 in 1983–1987 to 393 in 2008–2012. Moreover, 84 recorded populations in the long-standing CRs were listed in both VI and XI volumes (i.e, during 1983–1987 and 2008–2012, which is approximately 25-year span).

The volumes followed the evolution of the International Classification of Diseases (ICD), with BC coded according to the 9^th^ revision up to 1992 (ICD-9 = 174, all histologies), and according to the 10^th^ revision thereafter (ICD-10 = C50, all histologies). The collection of these datasets fully complied with international standards of cancer coding, and they were thus comparable across calendar years.

First, a cross-sectional analysis quantified the association between the median age at BC diagnosis and the median age of the corresponding population by including the 393 unique populations recorded in the XI volume (representing nearly one sixth of the world population located in 65 countries). The considered CRs were also analyzed by *a priori* selected geographical areas (Eastern Mediterranean and Africa, Asia, the Americas, Europe, and Oceania) according to the previously observed disparities of worldwide early BC onset^3,13^. To assess the consistency of the results, we repeated the analysis for five previous periods of registration (VI to X volumes). Second, a longitudinal analysis considering merely the 84 populations recorded in the long-standing CRs listed in both volumes VI and XI evaluated the influence of the 25-year population ageing on the age at onset of BC.

The structure of the resident population by age (quinquennia), sex, calendar period, and cancer registry was retrieved from the same publicly available IARC database of the cases.

### Statistical analyses

We broadly summarized for each CR the age composition of incident BC cases and the corresponding population structure by calculating the median age. The median age is the middle value dividing the population in two halves of equal size; it is an indicator that summarizes the age distribution of a population, i.e. higher the median, older the population^21,29,30^. In all IARC volumes, age is given in quinquennia of age (0–4, 5–9, …, 85+ years); thus, an approximated median was computed^31^. A sensitivity analysis was conducted to test for potential differences between the estimated and the observed median at BC onset. To this end, we extracted BC incidence for the period 2003–2007 (authorized by the CRs Directors: DS and HMC). For the Friuli Venezia Giulia CR, Italy (http://www.cro.sanita.fvg.it/it/azienda_informa/registro_tumori.html) the estimated median age was 64.6 years and the observed one was 64.7 years, whereas for the Setif CR, Algeria (www.ennour.org) the estimated median was 48.3 years and the observed one was 48.4 years.

The type of relationship between age at BC onset and age of the population was visually examined by means of a scatterplot. Thus, we assumed a linear shape between the two medians. The strength of association was quantified by means of a simple linear regression model of the least square method considering the age of the population as the predictor variable, and the age at BC onset as the dependent variable. The slope of the regression line estimated how much age at BC changes for one-year increase of the median age of the population. The r-squared (r^2^) indicated the percentage of the variance of age for BC that is explained through a linear relationship by the population ageing. The overall F-test was calculated to test if the r^2^ value was significantly different from zero. A model was calculated for each of the six calendar periods considered by including all the available CRs.

To support the strength of our study we carried out two sensitivity analyses. Firstly, although Editors of the Cancer in Five Continents verified and certified that all accepted datasets were of sufficiently high quality to merit their inclusion in the volumes, some datasets did not fully meet all criteria^32^ concerning quality and completeness of partly or all cancer sites, population or death certificates. Therefore, we recalculated the models for the 2008–2012 period excluding 109 cancer registries that did not fully meet the quality requirements (i.e, we analyzed 284 cancer registries instead of 393). Secondly, as it is known that age-specific rates of BC increase exponentially with age^33^, we verified whether an exponential relationship between age at BC onset and age of the population explained variance better than a linear model. For the period 2008–2012 the explained variances were 0.40 for the exponential model and 0.42 for the linear model. Therefore, the linear model was chosen for simplicity of graphical interpretation.

A 2-point line graph displayed the median age at BC according to the median age of the underlying population during the first (1983–1987) and last period (2008–2012) of observation in the 84 populations recorded in the long-standing CRs. Due to the peculiar pattern of the graph, a receiver operating characteristic (ROC) curve was calculated to identify the optimal cut-off of the median age for BC in 1983–1987 that best predicted its decreasing after 25-years^34^. Age-standardized incidence rates (ASR) (world population) for the 2008–2012 period were computed in early, intermediate, and late age at BC onset age groups (i.e, 20–44, 45–59, and 60–74 years), separately, for the three broad geographical areas selected *a priori*. For some CRs, data in older age groups (i.e. >75 years) were incomplete thus rates were not computed.

A p-value < 0.05 was considered statistically significant for the linear slope and r^2^. All statistical analyses were carried out using SAS (version 9.4 SAS Institute, Cary, NC, USA).

### Ethical approval

This article does not contain any studies with human participants or animals performed by any of the authors.

## Data Availability

The datasets analysed during the current study were downloaded from the IARC website: http://ci5.iarc.fr. And the currently analysed data are available from the corresponding author upon reasonable request.
